# Multimodal reasoning agent for enhanced ophthalmic decision-making: a preliminary real-world clinical validation

**DOI:** 10.3389/fcell.2025.1642539

**Published:** 2025-07-23

**Authors:** Yijing Zhuang, Dong Fang, Pengfeng Li, Bingyu Bai, Xiangqing Hei, Lujia Feng, Wangting Li, Shaochong Zhang

**Affiliations:** Shenzhen Eye Hospital, Shenzhen Eye Institute, Jinan University, Shenzhen, Guangdong, China

**Keywords:** artificial intelligence, large language models, reasoning agent, GPT-4o, ocular diseases

## Abstract

Although large language models (LLMs) show significant potential in clinical practice, accurate diagnosis and treatment planning in ophthalmology require multimodal integration of imaging, clinical history, and guideline-based knowledge. Current LLMs predominantly focus on unimodal language tasks and face limitations in specialized ophthalmic diagnosis due to domain knowledge gaps, hallucination risks, and inadequate alignment with clinical workflows. This study introduces a structured reasoning agent (ReasonAgent) that integrates a multimodal visual analysis module, a knowledge retrieval module, and a diagnostic reasoning module to address the limitations of current AI systems in ophthalmic decision-making. Validated on 30 real-world ophthalmic cases (27 common and 3 rare diseases), ReasonAgent demonstrated diagnostic accuracy comparable to ophthalmology residents (*β* = −0.07, *p* = 0.65). However, in treatment planning, it significantly outperformed both GPT-4o (*β* = 0.49, *p* = 0.01) and residents (*β* = 1.71, *p* < 0.001), particularly excelling in rare disease scenarios (all *p* < 0.05). While GPT-4o showed vulnerabilities in rare cases (90.48% low diagnostic scores), ReasonAgent’s hybrid design mitigated errors through structured reasoning. Statistical analysis identified significant case-level heterogeneity (diagnosis ICC = 0.28), highlighting the need for domain-specific AI solutions in complex clinical contexts. This framework establishes a novel paradigm for domain-specific AI in real-world clinical practice, demonstrating the potential of modularized architectures to advance decision fidelity through human-aligned reasoning pathways.

## 1 Introduction

The integration of artificial intelligence (AI) into ophthalmology has demonstrated transformative potential in automating image analysis (Feng et al., 2025; Rao et al., 2023), streamlining diagnostic workflows (Choi et al., 2024; Waisberg et al., 2024), and enhancing clinical decision-making (Delsoz et al., 2023; Tan et al., 2024). Numerous AI systems have been developed to tackle different tasks such as interpreting ophthalmic images, including fundus photography (Gulshan et al., 2016; Li et al., 2018), optical coherence tomography (OCT) (Schlegl et al., 2018), and scanning laser ophthalmoscopy (SLO) (Meyer et al., 2017; Tang et al., 2021), whose performance benchmarks often rival human experts in controlled settings. On the other hand, the advent of large language models (LLMs), particularly generative AI systems like ChatGPT, has rapidly expanded public access to AI technologies. Such models generate human-like responses from text prompts, offering applications ranging from facilitating physician-patient communication to synthesizing clinical data (Dave et al., 2023; Thirunavukarasu et al., 2023; Wu et al., 2023; Goh et al., 2024; Tangsrivimol et al., 2025; Yang X. et al., 2025). However, the diagnosis and management of many ophthalmic diseases require a complex integration of multimodal imaging interpretation and contextual clinical information (Yang et al., 2023; Gong et al., 2024). Current AI tools are predominantly designed for singular tasks and lack dynamic reasoning capabilities to emulate clinicians’ integrative decision-making processes (Homolak, 2023; Li et al., 2025; Wang et al., 2025). This critical gap limits their utility in real-world scenarios where diagnostic accuracy hinges on correlating heterogeneous data sources.

GPT-4o, OpenAI’s multimodal large language model, demonstrates enhanced capability in processing hybrid inputs (text, imaging, and audio) through cross-modal alignment (Shea et al., 2023). While this capability offers distinct advantages for analyzing medical data, the model’s multimodal architecture inadvertently amplifies hallucination risks, which may generate descriptions of pathological features absent from actual imaging findings (Shea and Ma, 2023; Chen D. et al., 2024; Günay et al., 2024; Li and Li, 2024). Furthermore, its black-box reasoning process fails to provide traceable diagnostic rationales anchored in medical literature, posing significant concerns for real-world clinical applications (Keles et al., 2025). Moreover, GPT-4o′s reliance on general-domain training data limits its mastery of specialized ophthalmic knowledge, particularly rare disease patterns and region-specific diagnostic criteria (Cai et al., 2024). Although retrieval-augmented generation (RAG) enhances LLM responses by retrieving relevant information from external sources before generating answers, improving accuracy and reducing hallucinations (Lewis et al., 2020; Nguyen et al., 2025; Song et al., 2025), conventional RAG systems exhibit critical shortcomings in ophthalmology applications: they frequently retrieve contextually irrelevant guidelines due to inadequate understanding of imaging biomarkers while mechanically concatenating retrieved evidence without synthesizing pathophysiological logic, resulting in clinically incoherent recommendations (Gargari and Habibi, 2025; Yang R. et al., 2025). This dual challenge of multimodal hallucination control and context-aware knowledge integration necessitates an architectural paradigm that synergistically combines the perceptual strengths of multimodal LLMs with rigorous evidence-based reasoning. To bridge these gaps, a structured framework that seamlessly integrates multimodal image analysis, real-time knowledge retrieval, and clinical reasoning is urgently needed.

In January 2025, DeepSeek introduced DeepSeek-R1, an innovative open-source reasoning LLM rapidly gaining worldwide prominence (Guo et al., 2025). Differing from opaque models, DeepSeek-R1 enables transparent, hierarchical reasoning through probabilistic causal graphs, dynamically resolving conflicting clinical evidence to produce auditable diagnostic pathways (Moëll et al., 2025; Sandmann et al., 2025). Additionally, its offline deployment capability allows healthcare institutions to locally operate and adapt the model without internet dependency, ensuring compliance with stringent data privacy regulations by eliminating sensitive data transmission, thereby fortifying security and confidentiality in clinical workflows (Sandmann et al., 2025).

Here, we proposed a structured reasoning agent (ReasonAgent) integrating three specialized modules: (1) a vision understanding module leveraging GPT-4o to analyze multimodal ophthalmic images and flag abnormalities; (2) a RAG module that retrieves diagnostic criteria from a curated knowledge base of ophthalmic guidelines based on patient history and exam findings; and (3) a diagnostic reasoning module (DeepSeek-R1) that synthesizes image interpretation, retrieved evidence, and clinical narratives to generate final diagnoses and treatment plans. To evaluate its clinical applicability, we compared the ReasonAgent’s performance against standalone GPT-4o outputs and answers from three ophthalmology residents across 30 real-world cases. This study aim to investigate whether a structured ReasonAgent can surpass general-purpose LLMs in ophthalmic diagnosis and evaluate how AI-assisted decision-making compares to human resident physicians in complex real-world scenarios.

## 2 Materials and methods

### 2.1 Study design and participants

This comparative, single-center, cross-sectional study adheres to the Strengthening the Reporting of Observational Studies in Epidemiology (STROBE) reporting guideline. A total of 30 deidentified ophthalmic cases (collected from January to March 2025) were included, with all protected health information rigorously encrypted. These cases were randomly selected from a database of Jinan University-affiliated Shenzhen Eye Hospital clinic, ensuring diversity in disease severity and presentation.

### 2.2 ReasonAgent implementation

**FIGURE 1:**
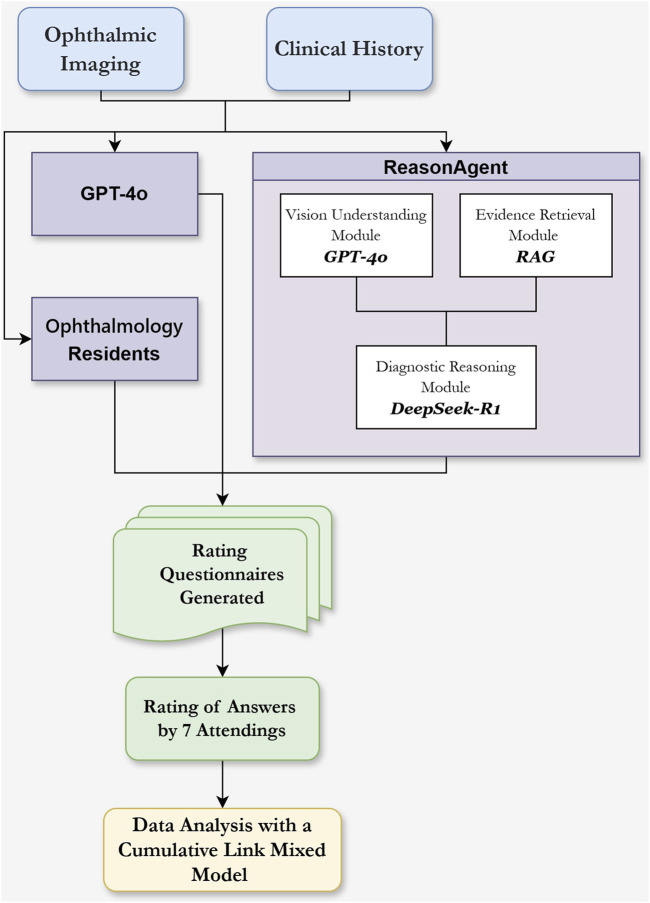
Flowchart of the Reasoning Agent Design and the Evaluation of Different Methods’ Responses in Clinical Ophthalmology Scenarios. Ophthalmic imaging (e.g., OCT, B scan, SLO, FFA) and clinical history serve as input sources. The Vision Understanding Module (GPT-4o) analyzes ophthalmic images for abnormalities and descriptions. The Evidence Retrieval Module (RAG) extracts diagnostic knowledge from guidelines based on clinical history and ocular examination. These outputs, combined with clinical history text, are input into the Diagnostic Reasoning Module (DeepSeek-R1) within the reasoning agent for diagnostic analysis and treatment planning. Comparison groups included standalone GPT-4o and three residents. Responses were evaluated using Likert scales by 7 attending physicians.

We developed a hierarchical ReasonAgent (Figure 1) through localized deployment of Dify. AI (Beijing, China, v1.0.0) workflow orchestration platform, where GPT-4o, RAG architecture, and DeepSeek-R1 were programmatically chained as core processing nodes. We configured all system components through dedicated API interfaces, establishing automated data pipelines between modules. The agent was programmed to emulate clinical ophthalmologists’ diagnostic workflow through the following technical implementation.

#### 2.2.1 Vision understanding module

GPT-4o (OpenAI, USA; version 2024–11–20, temperature = 0.7) was adopted as a visual analysis module. This module received multimodal ophthalmic imaging inputs (e.g., OCT, B-scan, SLO, fluorescein fundus angiography/indocyanine green angiography (FFA/ICGA)) with the prompt provided in Supplementary Appendix 1.

#### 2.2.2 Evidence retrieval module

We employed a RAG architecture BGE-M3 (Chen J. et al., 2024) embeddings (designed by BAAI, China; provided by SiliconFlow, China) for multilingual knowledge retrieval. The knowledge base integrated two principal corpora: 1) Kanski’s Clinical Ophthalmology (ninth Edition) as foundational textbook knowledge, and 2) annually updated clinical guidelines (January 2024 to February 2025) from the American Academy of Ophthalmology (AAO) and the Chinese Medical Association (CMA). A unified prompting framework was adopted across retrieval components, synchronizing with the DeepSeek-R1 model through the shared instruction.

#### 2.2.3 Diagnostic reasoning module

We applied DeepSeek-R1 (DeepSeek, China; 671B version, temperature = 0.6) for comprehensive reasoning analysis. The model received formatted inputs: [Imaging Analysis] + [Retrieved Evidence] + [Clinical History], generating a reasoning process, preliminary diagnosis, and treatment plans with explicit citations, with the detailed prompt provided in Supplementary Appendix 1.

### 2.3 Case selection

To evaluate the models’ performance versus clinicians across diseases of varying complexity, we curated 30 clinical cases spanning corneal diseases, cataracts, glaucoma, and fundus disorders. The cohort included 27 common ophthalmic conditions (e.g., age-related cataracts, retinal detachment) and 3 rare diseases (Coats disease, malignant glaucoma, Vogt-Koyanagi-Harada syndrome).

### 2.4 Comparison and scoring criteria

To establish comparative benchmarks, clinical cases in Chinese with associated imaging data were independently analyzed using three methods: 1) the output of ReasonAgent pipeline, 2) GPT-4o analysis with explicit instructions shown in Supplementary Appendix 1, 3) three residents producing comprehensive diagnoses and prioritized treatment plans.

The diagnoses and treatment plans generated by ReasonAgent, GPT4o, and residents were anonymized and randomly presented to the panel of 7 senior attending physicians for evaluation using a 5-point Likert scale:

1: Unacceptably poor or containing critical errors
2: Poor accuracy with potentially harmful errors/omissions
3: Neutral (moderate quality with ambiguous/minor issues)
4: Good quality with non-critical errors/omissions
5: Excellent quality with no errors/omissions

### 2.5 Statistical analysis

Descriptive statistics were reported as means, standard deviations (SD), along with medians and interquartile ranges (IQR) of the Likert scores. A Cumulative Link Mixed Model (CLMM) fitted with the Laplace approximation was implemented to evaluate decision-making performance differences between methods (ReasonAgent, GPT-4o, and residents). Two separate model analyses were conducted for diagnoses and treatment plans, with each model preserving identical random effects structures. Fixed effects were modeled to assess the accuracy of diagnoses and treatment plans, while random effects accounted for variability across individual cases and between different raters. Main analytical indices included estimated marginal means (EMMs) with 95% confidence interval (CI), and interpretation of intraclass correlation coefficients (ICCs) derived from variance components of the logistic distribution to quantify proportional variance contributions of case-level and rater-level heterogeneity, with lower ICC values indicating higher consistency. Post hoc pairwise comparisons with Tukey adjustment for multiple testing were conducted to identify specific group differences. For subgroup comparisons between common and rare diseases, Kruskal–Wallis tests were performed to detect overall differences in scores across groups within each disease category. Dunn’s *post hoc* tests with Bonferroni adjustment were applied for pairwise group comparisons. Additionally, low-score proportions (scores≤2) were analyzed as a secondary metric to evaluate performance across methods. The level of significance was set at *p* < 0.05. All analyses were conducted in R (version 4.4.3) with the ordinal package (*clmm* function) for model fitting, *lme4* for mixed-effects infrastructure, *emmeans* for marginal mean estimation and comparisons, and *FSA* for non-parametric subgroup analysis.

## 3 Results

### 3.1 Fixed effects analysis of reasoning performance across methods

**FIGURE 2:**
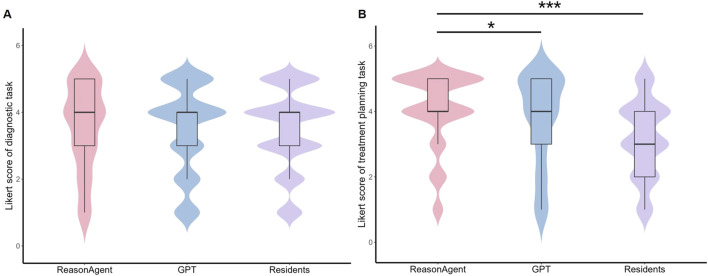
Distribution of Likert Scores for Different Methods in Diagnostic Tasks and Treatment Planning Tasks. **(A)** Violin plot of Likert scores for diagnostic tasks; **(B)** Violin plot of Likert scores for treatment planning tasks. Embedded boxplots illustrate the interquartile range (25th to 75th percentile), the median (black horizontal line), and the whiskers represent the range of scores excluding outliers. Statistical analysis revealed no significant differences in diagnostic task scores between ReasonAgent, GPT-4o, and residents. In contrast, treatment planning tasks showed significantly higher scores for ReasonAgent than GPT-4o and residents. *p < 0.05, **p < 0.01, ***p < 0.001.

The CLMM analysis revealed no statistically significant differences in diagnostic reasoning performance between the ReasonAgent (median = 4, IQR = 3–5), GPT-4o (median = 4, IQR = 3–4), and residents (median = 4, IQR = 3–4, Figure 2A). Fixed effects comparisons showed non-significant deviations for GPT-4o (*β* = 0.04, 95% CI: −0.31 to 0.40, *p* = 0.81) and physicians (*β* = −0.07, 95% CI: −0.36 to 0.22, *p* = 0.65, Table 1) relative to the ReasonAgent. Post hoc pairwise contrasts revealed consistently non-significant differences across all groups (Table 1). These findings demonstrate concordant diagnostic reasoning across methods, with algorithmic approaches (ReasonAgent and GPT-4o) achieving performance comparable to human physicians.

**TABLE 1:** Fixed effects analysis and post-hoc pairwise comparisons of different methods.

Group	EMMs	OR (95% CI)	*z*-value	*p*-value^a^	Adjusted *p*-value^b^
Diagnostic task					
ReasonAgent (reference)	0	1.00	—	—	vs. GPT-4o (0.97)
GPT-4o	0.04	1.05 (0.73–1.49)	0.24	0.81	vs. Resident (0.73)
Resident	−0.07	0.94 (0.70–1.25)	−0.45	0.65	vs. ReasonAgent (0.90)
Treatment planning task					
ReasonAgent (reference)	0	1.00	—	—	vs. GPT-4o (0.03)*
GPT-4o	0.05	0.62 (0.42–0.89)	−2.54	0.011*	vs. Resident (<0.001)***
Resident	−1.71	0.94 (0.13–0.25)	−10.69	<0.001***	vs. ReasonAgent (<0.001)***

EMMs: Estimated Marginal Means, OR: odds ratio, CI: confidence interval.

^a^ Cumulative Link Mixed Model (CLMM) fitted with the Laplace approximation.

^b^ Post-hoc pairwise comparisons with Tukey adjustment.

*p < 0.05, **p < 0.01, ***p < 0.001.

In terms of the treatment planning task, the statistical analysis identified significant between-group differences across methods. Fixed effects analysis revealed that ReasonAgent (median = 4, IQR = 4–5) significantly outperformed both GPT-4o (median = 4, IQR = 3-5, *β* = 0.49, 95% CI: −0.86 to −0.11, *p* = 0.01) and residents (median = 3, IQR = 2-4, *β* = 1.71, 95% CI: −2.03 to −1.40, *p* < 0.001, Table 1; Figure 2B). Post-hoc comparisons with Tukey adjustment demonstrated significantly superior performance of Reasoning Agent over both GPT-4o (*β* = 0.486, *p* = 0.030) and residents (*β* = 1.714, *p* < 0.001, Table 1). GPT-4o also demonstrated significant advantages over human physicians (*β* = 1.228, *p* < 0.001). These results indicate that algorithmic approaches (Reasoning Agent and GPT-4o) consistently outperformed human physicians in treatment plan formulation, with ReasonAgent achieving particularly enhanced efficacy relative to GPT-4o.

### 3.2 Rater-level variability analysis

Low variability in rating stringency was observed across raters, with the random intercept variance between raters in the diagnostic reasoning task estimated at σ^2^ = 0.40 (SD = 0.63, Table 2). This between-rater heterogeneity corresponded to an ICC of 0.08 (95% CI: 0–0.16), indicating that approximately 8% of the total variance originated from systematic differences in scoring severity across raters. This indicates that low inter-rater disagreement in Likert scores for diagnostic tasks, reflecting relatively consistent clinical expertise or interpretive standards among raters.

**TABLE 2:** Random effects analysis in diagnostic and treatment planning tasks.

Group	Variance	SD	Groups	ICC (95% CI)
Diagnostic task				
Case	1.45	1.20	30	0.28 (0.18–0.39)
Rater	0.40	0.63	7	0.08 (0–0.16)
Treatment planning task				
Case	1.01	1.01	30	0.21 (0.15–0.31)
Rater	0.47	0.68	7	0.10 (0.04–0.18)

SD, standard deviation, ICC, intraclass correlation coefficient.

While in the treatment planning task, the variability of rating is slightly increased compared to the diagnostic task (σ^2^ = 0.47, SD = 0.68, Table 2), and the corresponding ICC is 0.10 (95% CI: 0.04–0.18), suggesting greater inconsistency in treatment planning assessment ratings.

### 3.3 Case-level variability analysis

In the diagnostic reasoning task, considerable case-level heterogeneity was observed, with case-level random intercepts accounting for significant variance in diagnostic performance ratings (σ^2^ = 1.44, SD = 1.20, Table 2). The ICC confirmed that 28% (ICC = 0.28, 95% CI: 0.18–0.39)of total variance stemmed from systematic differences between clinical cases. This indicates that clinical characteristics or case complexity exerted a notable influence on diagnostic assessments. For the treatment planning task, while case-level variability remained significant (σ^2^ = 1.01, SD = 1.01, Table 2), its absolute contribution decreased (ICC = 0.21,95% CI: 0.15–0.31), with the relative contribution to total variance components also reducing to 68.5% compared to diagnostic tasks.

Pronounced performance variations existed between rare and common cases across methods. While all three approaches achieved comparable ratings for common cases (ReasonAgent: median = 4, IQR = 3-5, GPT-4o: median = 4, IQR = 3-5, residents: median = 4, IQR = 3–4; all *p* > 0.05, Table 3), rare cases revealed substantial divergence in diagnostic performance. GPT-4o demonstrated significantly lower performance (median = 1, IQR = 1–2) compared to ReasonAgent (median = 3, IQR = 1-4, *p* = 0.02) and residents (median = 3, IQR = 1-4, *p* = 0.03, Table 3). This substantial gap persisted despite limited rare-case samples (n = 3), reflecting methodological vulnerabilities. In addition, GPT-4o yielded low scores (≤2) in 90.48% of rare-case diagnostic tasks, a proportion substantially surpassing the rates recorded for ReasonAgent (38.10%) and physicians (49.21%), suggesting greater vulnerability to the diagnostic complexity of rare ophthalmic conditions.

**TABLE 3:** Diagnostic and treatment planning performance by method and case rarity.

Group	Method	Mean score ±SD	Median score (IQR)	% low scores (≤2)	Pairwise Comparisons (*p*-values^a^)
Diagnostic task					
Common diseases	ReasonAgent	3.59 ± 1.27	4 (3–5)	39/189 (15.34%)	vs. GPT-4o (>0.99)
	GPT-4o	3.68 ± 1.18	4 (3–5)	32/189 (16.93%)	vs. Resident (0.47)
	Resident	3.57 ± 1.16	4 (3–4)	100/567 (17.64%)	vs. ReasonAgent (>0.99)
Rare diseases	ReasonAgent	2.91 ± 1.58	3 (1–4)	8/21 (38.10%)	vs. GPT-4o (0.02)*
	GPT-4o	1.67 ± 0.91	1 (1–2)	19/21 (90.48%)	vs. Resident (0.03)*
	Resident	2.60 ± 1.40	3 (1–4)	31/63 (49.21%)	vs. ReasonAgent (>0.99)
Treatment planning task					
Common diseases	ReasonAgent	4.12 ± 1.10	4 (4–5)	21/189 (11.11%)	vs. GPT-4o (0.67)
	GPT-4o	3.96 ± 1.23	4 (3–5)	29/189 (15.34%)	vs. Resident (<0.001)***
	Resident	3.27 ± 1.11	3 (3–4)	139/567 (24.51%)	vs. ReasonAgent (<0.001)***
Rare diseases	ReasonAgent	3.10 ± 1.30	4 (2–4)	8/21 (38.10%)	vs. GPT-4o (<0.001)***
	GPT-4o	1.67 ± 1.11	1 (1–2)	17/21 (80.95%)	vs. Resident (0.14)
	Resident	2.25 ± 1.23	2 (1–3)	39/63 (61.90%)	vs. ReasonAgent (0.04)*

SD, standard deviation, IQR, interquartile range.

^a^ Dunn’s post hoc tests with Bonferroni adjustment.

*p < 0.05, **p < 0.01, ***p < 0.001.

In the treatment planning task, we also observed distinct performance variations among the three methods in both common and rare diseases. For common cases, the algorithmic methods (ReasonAgent (median = 4, IQR = 4-5, *p* < 0.001) and GPT4o (median = 4, IQR = 3-5, *p* < 0.001)) demonstrated statistically significant superior scores compared to residents (median = 3, IQR = 3–4, Table 3), while showing no significant performance difference between the two algorithms methods (*p* = 0.67). In rare disease scenarios, ReasonAgent (median = 4, IQR = 2–4) significantly outperformed both GPT4o (median = 1, IQR = 1-2, *p* < 0.001, Table 3) and residents (median = 2, IQR = 1-3, *p* = 0.04), with GPT4o showing no advantage over human physicians (*p* = 0.14). Additionally, statistical analysis of low-performance probabilities (scores ≤2) corroborated these trends: For common disease treatment planning, both GPT4o (15.34%) and ReasonAgent (11.11%) exhibited significantly lower rates of low scores compared to human physicians (24.51%). And for rare diseases, ReasonAgent (38.10%) maintained a substantially lower low-score rate than both residents (61.90%) and GPT4o (80.95%), demonstrating its dual advantage in treatment planning for both common and rare clinical conditions.

## 4 Discussion

With the rapid development of large language models, artificial intelligence has demonstrated tremendous potential for application in the medical field. However, the diagnosis and treatment decision-making of ophthalmic diseases possess unique complexity: it not only relies on the meticulous interpretation of multimodal images but also requires the integration of heterogeneous data, including the current medical history and systemic comorbidities, to formulate personalized treatment plans. This study constructs an ophthalmic reasoning agent by integrating the modules of visual understanding, knowledge retrieval, and causal reasoning, and evaluates its performance in real ophthalmic cases.

Using 30 ophthalmic cases, we conducted a comprehensive performance evaluation of diagnostic reasoning and treatment planning across three methods: ReasonAgent, GPT-4o, and human physicians. The results demonstrated that in diagnostic tasks, the algorithmic methods (Reasoning Agent and GPT-4o) achieved performance comparable to ophthalmology residents. In treatment planning tasks, both algorithmic approaches significantly outperformed human doctors, with Reasoning Agent showing a notably superior performance compared to GPT-4o alone. One possible explanation is that diagnostic classification critically relies on quantifiable biomarkers, such as the foveal thickness in OCT. Both AI systems and human physicians can achieve this through pattern recognition. However, in treatment planning, the Reasoning Agent mitigates inexperience-driven cognitive biases among ophthalmology residents through evidence integration via RAG. Additionally, it can correlate influential features with the latest guidelines, thus circumventing the generalization errors of GPT-4o. This indicates that through the structured reasoning involving multimodal data integration and evidence anchoring, AI has the potential to transcend the limitations of a single model. Specifically, the visual module (GPT-4o) of the Reasoning Agent accurately captures imaging abnormalities, the RAG module retrieves the latest guidelines in real time, and the DeepSeek-R1 reasoning module strings together clinical information based on causal logic to form a traceable decision-making path. For instance, in the case of Coats’ disease, GPT-4o misdiagnosed it as retinoblastoma, and some residents confused it with persistent hyperplastic primary vitreous (PHPV). However, Reasoning Agent accurately identified the typical vascular abnormalities through the cross-validation of imaging features and guideline criteria, and through the clinical characteristics of the medical history, it gave the reasoning process of the diagnosis and avoided serious misdiagnosis. In a case of diabetic retinopathy, GPT-4o had a conflict in the identification of OCTA and B-scan images (OCTA detected tractional retinal detachment, while B-scan indicated “no characteristic strong echo signals of retinal detachment, suggesting that the retina is attached”). However, the reasoning module (DeepseekR1) resolved this conflict, presented a detailed thought process, and obtained the correct diagnosis and treatment plan. On the other hand, DeepSeek-R1 demonstrates superior capabilities in processing clinical documentation in Chinese. Since this study is based on medical records in Chinese, its language architecture can more accurately capture the key clinical features in the Chinese context and reduce the ambiguity caused by direct Chinese-English translation in term mapping. These findings highlight the necessity of domain-specific architectures to constrain general model limitations while augmenting human expertise. These results are consistent with the theory proposed by Bommasani et al. that “clinical AI needs to integrate perception and reasoning”, suggesting that future system designs should give priority to cross-modal alignment and evidence-based constraint mechanisms (Bommasani et al., 2021).

The observed case-level heterogeneity (intraclass correlation coefficient for diagnosis, ICC = 0.28, P < 0.01) may reflect limitations of general large language models in processing multimodal inputs with varying pathological complexities. While GPT-4o′s unstructured reasoning suffices for common conditions with typical patterns, its failure in rare cases (90.48% low diagnostic scores) suggests insufficient domain-specific knowledge acquisition and overreliance on probabilistic associations rather than pathophysiological logic. ReasonAgent’s superior performance in rare disease diagnosis likely stems from its hybrid architecture: a vision-language model enables lesion localization, while RAG constrains reasoning to evidence-based diagnostic pathways, compensating for the scarcity of low-prevalence disease patterns in general training data. In treatment planning, the ReasonAgent’s advantage over both GPT-4o and resident physicians indicates that structured knowledge retrieval mitigates knowledge gaps or clinical experience deficits in junior doctors. GPT-4o′s poorer therapeutic performance compared to diagnosis aligns with its lack of hierarchical treatment action structures—a critical gap addressed by RAG, which prioritizes guideline-recommended interventions. These findings highlight the utility of hybrid AI systems integrating deep visual understanding with evidence-based reasoning in overcoming the limitations of general-purpose models in complex medical scenarios.

ReasonAgent’s hierarchical architecture distinguishes it from a single black-box large model like GPT-4o. By modularizing clinical reasoning into discrete stages—imaging analysis, knowledge retrieval, biometric validation, and evidence-based conclusion generation—it is analogous to the systematic logic of human clinicians while preserving traceable reasoning paths. Throughout this process, a reasoning path visualization module preserves the full decision-making trajectory, enabling clinicians to retrace critical nodes such as biological parameter calculations and literature evidence citations. This feature distinctly differentiates ReasonAgent from general models, whose untraceable probabilistic output paradigms prevent the attribution of diagnostic or therapeutic suggestions to specific evidential sources. Therefore, in clinical applications, the Reasoning Agent has two major application potentials. Firstly, as a decision-support tool for junior doctors and primary care doctors, its traceable reasoning process facilitates rapid and accurate identification of evidence-based rationales for therapeutic plans, addressing knowledge gaps in less experienced clinicians. Secondly, as a quality control tool, it can serve as an auxiliary reference for junior doctors in medical record writing, reducing the risks of missed or misdiagnosis. Its evidence-traceable reasoning framework can also act as an assistant in the medical record systems to assist in systematic validation of medical records.

In this study, several critical observations concerning large language models (LLMs) merit attention. Firstly, RAG cannot fully resolve hallucinations (Gargari and Habibi, 2025; Yang R. et al., 2025), such as mis-defining normative biometric thresholds (e.g., foveal thickness ranges) and conflating diagnostic criteria (e.g., high myopia). These errors emphasize the need for real-time biometric verification to counter parameter fabrication tendencies. Secondly, the GPT-4o demonstrated elevated misjudgment rates for highly specialized imaging data, such as anterior segment photography or dynamically interpreted datasets like B-scan, errors included misidentifying corneal reflection points as corneal leukomas and failing to determine posterior movement positivity in B-scans. Such misinterpretations propagate downstream analytical errors in Deepseek-R1 (e.g., the epiretinal membrane case unrecognized by GPT-4o in this study). These observations accentuate the imperative for the development of ophthalmology-specific visual processing modules, which transcend the limitations of generic image recognition algorithms. Futhermore, deploying AI-driven reasoning agents in clinical settings requires careful attention to data privacy, infrastructure, human oversight, and ethics. The proposed method is built upon closed-source services for preliminary clinical validation. To address patient privacy concerns in clinical practice, these components can be replaced with other locally deployed open-source models. However, local deployment incurs substantially higher costs and operational overhead. For example, deploying a large language model of DeepSeek R1’s scale (671 billion parameters) poses significant challenges for hospital infrastructure stability and maintenance. Besides, although our method has demonstrated diagnostic capabilities on par with those of junior human clinicians and even superior performance in formulating treatment plans, it is intended solely as a decision-support tool and cannot replace human clinicians. In certain scenarios, the performance may also reflect biases originating from the training data and the models themselves. Beyond model-specific limitations, the current study still has several methodological limitations. The relatively modest sample size of 30 cases, with rare diseases constituting only 10% (n = 3), may compromise the statistical power required for comprehensive subgroup analyses, and the inter-case heterogeneity inherent in small cohorts may obscure statistically significant differences in diagnostic performance across methods. Future investigations should endeavor to expand the sample cohort to robustly validate the stability of ReasonAgent. Additionally, the small sample of three resident evaluators may introduce observer bias; future studies should include senior ophthalmologists to enhance validation rigor.

This study developed an ophthalmic ReasonAgent integrating visual understanding (GPT-4o), evidence retrieval (RAG), and diagnostic reasoning (DeepSeek-R1) modules to enable interpretable decision-making in multimodal clinical scenarios. Testing on 30 real-world cases demonstrated that the ReasonAgent exhibited diagnostic accuracy comparable to that of resident ophthalmologists, while significantly outperforming both human physicians and the general-purpose large language model GPT-4o in treatment planning. Its core advantage lies in a hierarchical reasoning mechanism: dynamic knowledge retrieval is triggered by imaging feature analysis, combined with causal logic to generate traceable diagnostic and therapeutic decision trees. This approach mitigates GPT-4o′s cross-modal misalignment and reduces empirical biases inherent to resident physicians in complex cases. The ReasonAgent addresses the cross-modal alignment limitations of general LLMs and offers evidence-based reasoning outcomes, establishing a novel framework for the application of medical artificial intelligence in real-world clinical practice.

## Data Availability

The original contributions presented in the study are included in the article/Supplementary Material, further inquiries can be directed to the corresponding author.

## References

[B1] Bommasani R. Hudson D. Adeli E. Altman R. Arora S. Arx S. (2021). On the opportunities and risks of foundation models.

[B2] Cai X. Zhan L. Lin Y. (2024). Assessing the accuracy and clinical utility of GPT-4O in abnormal blood cell morphology recognition. Digit. Health 10, 20552076241298503. 10.1177/20552076241298503 39502485 PMC11536573

[B3] Chen D. Huang R. S. Jomy J. Wong P. Yan M. Croke J. (2024a). Performance of multimodal artificial intelligence chatbots evaluated on clinical oncology cases. JAMA Netw. Open 7 (10), e2437711. 10.1001/jamanetworkopen.2024.37711 39441598 PMC11581577

[B4] Chen J. Xiao S. Zhang P. Luo K. Lian D. Liu Z. J. a.p.a. (2024b). Bge m3-embedding: multi-Lingual, multi-functionality, multi-granularity text embeddings through self-knowledge distillation.

[B5] Choi J. Oh A. R. Park J. Kang R. A. Yoo S. Y. Lee D. J. (2024). Evaluation of the quality and quantity of artificial intelligence-generated responses about anesthesia and surgery: using ChatGPT 3.5 and 4.0. Front. Med. 11, 1400153. 10.3389/fmed.2024.1400153 PMC11269144 39055693

[B6] Dave T. Athaluri S. A. Singh S. (2023). ChatGPT in medicine: an overview of its applications, advantages, limitations, future prospects, and ethical considerations. Front. Artif. Intell. 6, 1169595. 10.3389/frai.2023.1169595 37215063 PMC10192861

[B7] Delsoz M. Raja H. Madadi Y. Tang A. A. Wirostko B. M. Kahook M. Y. (2023). The use of ChatGPT to assist in diagnosing glaucoma based on clinical case reports. Ophthalmol. Ther. 12 (6), 3121–3132. 10.1007/s40123-023-00805-x 37707707 PMC10640454

[B8] Feng S. Yang J. Zhao X. Zhao J. Du Y. Yu W. (2025). Assessment of synthetic post-therapeutic OCT images using the generative adversarial network in patients with macular edema secondary to retinal vein occlusion. Front. Cell Dev. Biol. 13, 1609567. 10.3389/fcell.2025.1609567 40535568 PMC12174594

[B9] Gargari O. K. Habibi G. (2025). Enhancing medical AI with retrieval-augmented generation: a mini narrative review. Digit. Health 11, 20552076251337177. 10.1177/20552076251337177 40343063 PMC12059965

[B10] Goh E. Gallo R. Hom J. Strong E. Weng Y. Kerman H. (2024). Large language model influence on diagnostic reasoning: a randomized clinical trial. JAMA Netw. Open 7 (10), e2440969. 10.1001/jamanetworkopen.2024.40969 39466245 PMC11519755

[B11] Gong D. Li W.-T. Li X.-M. Wan C. Zhou Y.-J. Wang S.-J. (2024). Development and research status of intelligent ophthalmology in China. Int. J. Ophthalmol. 17 (12), 2308–2315. 10.18240/ijo.2024.12.20 39697896 PMC11589450

[B12] Gulshan V. Peng L. Coram M. Stumpe M. C. Wu D. Narayanaswamy A. (2016). Development and validation of a deep learning algorithm for detection of diabetic retinopathy in retinal fundus photographs. jama 316 (22), 2402–2410. 10.1001/jama.2016.17216 27898976

[B13] Günay S. Öztürk A. Yiğit Y. (2024). The accuracy of gemini, GPT-4, and GPT-4o in ECG analysis: a comparison with cardiologists and emergency medicine specialists. Am. J. Emerg. Med. 84, 68–73. 10.1016/j.ajem.2024.07.043 39096711

[B14] Guo D. Yang D. Zhang H. Song J. Zhang R. Xu R. (2025). Deepseek-r1: incentivizing reasoning capability in llms *via* reinforcement learning.

[B15] Homolak J. (2023). Opportunities and risks of ChatGPT in medicine, science, and academic publishing: a modern Promethean dilemma. Croat. Med. J. 64 (1), 1–3. 10.3325/cmj.2023.64.1 36864812 PMC10028563

[B16] Keles A. Illeez O. G. Erbagci B. Giray E. (2025). Artificial intelligence-generated responses to frequently asked questions on coccydynia: evaluating the accuracy and consistency of GPT-4o's performance. Arch. Rheumatol. 40 (1), 63–71. 10.46497/ArchRheumatol.2025.10966 40264482 PMC12010271

[B17] Lewis P. Perez E. Piktus A. Petroni F. Karpukhin V. Goyal N. (2020). Retrieval-augmented generation for knowledge-intensive nlp tasks. Adv. neural Inf. Process. Syst. 33, 9459–9474. 10.48550/arXiv.2005.11401

[B18] Li Q. Li P. H. (2024). Transformative potential of GPT-4o in clinical immunology and allergy: opportunities and challenges of real-time voice interaction. Asia Pac Allergy 14 (4), 232–233. 10.5415/apallergy.0000000000000152 39624445 PMC11608632

[B19] Li Z. He Y. Keel S. Meng W. Chang R. T. He M. (2018). Efficacy of a deep learning system for detecting glaucomatous optic neuropathy based on color fundus photographs. Ophthalmology 125 (8), 1199–1206. 10.1016/j.ophtha.2018.01.023 29506863

[B20] Li Z. Wang Z. Xiu L. Zhang P. Wang W. Wang Y. (2025). Large language model-based multimodal system for detecting and grading ocular surface diseases from smartphone images. Front. Cell Dev. Biol. 13, 1600202. 10.3389/fcell.2025.1600202 40486905 PMC12141289

[B21] Meyer M. I. Costa P. Galdran A. Mendonça A. M. Campilho A. (2017). “A deep neural network for vessel segmentation of scanning laser ophthalmoscopy images,” in Lecture notes in computer science (Springer International Publishing).

[B22] Moëll B. Sand Aronsson F. Akbar S. (2025). Medical reasoning in LLMs: an in-depth analysis of DeepSeek R1. Front. Artif. Intell. 8, 1616145–2025. 10.3389/frai.2025.1616145 40607450 PMC12213874

[B23] Nguyen Q. N. Nguyen A. D. Dang K. Liu S. Nguyen K. Wang S. Y. (2025). Advancing question-answering in ophthalmology with retrieval-augmented generation (RAG): benchmarking open-source and proprietary large language models. Investigative Ophthalmol. and Vis. Sci. 66 (8), 4638. 10.1101/2024.11.18.24317510

[B24] Rao A. Kim J. Kamineni M. Pang M. Lie W. Dreyer K. J. (2023). Evaluating GPT as an adjunct for radiologic decision making: GPT-4 *versus* GPT-3.5 in a breast imaging pilot. J. Am. Coll. Radiol. 20 (10), 990–997. 10.1016/j.jacr.2023.05.003 37356806 PMC10733745

[B25] Sandmann S. Hegselmann S. Fujarski M. Bickmann L. Wild B. Eils R. (2025). Benchmark evaluation of DeepSeek large language models in clinical decision-making. Nat. Med. 10.1038/s41591-025-03727-2 PMC12353792 40267970

[B26] Schlegl T. Waldstein S. M. Bogunovic H. Endstraßer F. Sadeghipour A. Philip A.-M. (2018). Fully automated detection and quantification of macular fluid in OCT using deep learning. Ophthalmology 125 (4), 549–558. 10.1016/j.ophtha.2017.10.031 29224926

[B27] Shea Y. F. Lee C. M. Y. Ip W. C. T. Luk D. W. A. Wong S. S. W. (2023). Use of GPT-4 to analyze medical records of patients with extensive investigations and delayed diagnosis. JAMA Netw. Open 6 (8), e2325000. 10.1001/jamanetworkopen.2023.25000 37578798 PMC10425828

[B28] Shea Y. F. Ma N. C. (2023). Limitations of GPT-4 in analyzing real-life medical notes related to cognitive impairment. Psychogeriatrics 23 (5), 885–887. 10.1111/psyg.13002 37366006 PMC11577998

[B29] Song S. Peng K. Wang E. Liu T. Y. A. (2025). Enhancing large language model performance on ophthalmology board-style questions with retrieval-augmented generation. Investigative Ophthalmol. and Vis. Sci. 66 (8), 3930.

[B30] Tan D. N. H. Tham Y.-C. Koh V. Loon S. C. Aquino M. C. Lun K. (2024). Evaluating chatbot responses to patient questions in the field of glaucoma. Front. Med. 11, 1359073. 10.3389/fmed.2024.1359073 PMC11267485 39050528

[B31] Tang F. Luenam P. Ran A. R. Quadeer A. A. Raman R. Sen P. (2021). Detection of diabetic retinopathy from ultra-widefield scanning laser ophthalmoscope images: a multicenter deep learning analysis. Ophthalmol. Retina 5 (11), 1097–1106. 10.1016/j.oret.2021.01.013 33540169

[B32] Tangsrivimol J. A. Darzidehkalani E. Virk H. U. H. Wang Z. Egger J. Wang M. (2025). Benefits, limits, and risks of ChatGPT in medicine. Front. Artif. Intell. 8, 1518049–2025. 10.3389/frai.2025.1518049 39949509 PMC11821943

[B33] Thirunavukarasu A. J. Ting D. S. J. Elangovan K. Gutierrez L. Tan T. F. Ting D. S. W. (2023). Large language models in medicine. Nat. Med. 29 (8), 1930–1940. 10.1038/s41591-023-02448-8 37460753

[B34] Waisberg E. Ong J. Masalkhi M. Zaman N. Sarker P. Lee A. G. (2024). GPT-4 to document ophthalmic post-operative complications. Eye (Lond) 38 (3), 414–415. 10.1038/s41433-023-02731-5 37714992 PMC10858225

[B35] Wang Y. Yang S. Zeng C. Xie Y. Shen Y. Li J. (2025). Evaluating the performance of ChatGPT in patient consultation and image-based preliminary diagnosis in thyroid eye disease. Front. Med. (Lausanne) 12, 1546706. 10.3389/fmed.2025.1546706 40041459 PMC11876178

[B36] Wu G. Lee D. A. Zhao W. Wong A. Sidhu S. (2023). ChatGPT: is it good for our glaucoma patients? Front. Ophthalmol. (Lausanne) 3, 1260415. 10.3389/fopht.2023.1260415 38983063 PMC11182305

[B37] Yang R. Ning Y. Keppo E. Liu M. Hong C. Bitterman D. S. (2025a). Retrieval-augmented generation for generative artificial intelligence in health care. npj Health Syst. 2 (1), 2. 10.1038/s44401-024-00004-1 PMC13354210 42527447

[B38] Yang W.-H. Shao Y. Xu Y.-W. Expert Workgroup of Guidelines on Clinical Research Evaluation of Artificial Intelligence in Ophthalmology 2023, Ophthalmic Imaging and Intelligent Medicine Branch of Chinese Medicine Education Association, Intelligent Medicine Committee of Chinese Medicin e Education Association (2023). Guidelines on clinical research evaluation of artificial intelligence in ophthalmology (2023). Int. J. Ophthalmol. 16 (9), 1361–1372. 10.18240/ijo.2023.09.02 37724285 PMC10475621

[B39] Yang X. Xu J. Ji H. Li J. Yang B. Wang L. J. F. i.O. (2025b). Early prediction of colorectal adenoma risk: leveraging large-language model for clinical electronic medical record data. Front. Oncol. 15, 1508455. 10.3389/fonc.2025.1508455 40444092 PMC12119310

