# Supplementary material for: Multimodal reasoning agent for enhanced ophthalmic decision-making: a preliminary real-world clinical validation
Source: Front Cell Dev Biol. 2025 Jul 23;13:1642539. doi: 10.3389/fcell.2025.1642539 (PMC12325206; doi:10.3389/fcell.2025.1642539)
Supplement: Supplementary file 1 [file Table1.docx]

**Appendix 1. Model details**

1. **Prompts of ReasonAgent:**
   1. Vision Understanding Module (GPT-4o):

This series contains [imaging modality, e.g., OCT] examinations of the patient's [laterality, e.g., left] eye. Provide a comprehensive characterization integrating cross-sectional imaging data, with particular attention to quantitative biomarkers where available. For macular assessments, explicitly reference central foveal thickness measurements as indicated by calibration scale markers.

- 1. Diagnostic Reasoning Module（Deepseek-R1）：

The following is a patient's medical record. Based on the patient's medical history, ocular examination findings, and imaging modalities, please provide comprehensive preliminary diagnoses (including but not limited to pseudophakia, refractive errors, systemic diseases, etc., using ICD-11 international classification codes). Subsequently, formulate a prioritized management plan for the patient.

Special notes: The descriptive interpretations of imaging data are derived from GPT-4o's multimodal analytical capabilities for image recognition, which can be considered reliable for observational descriptions but should not be adopted for diagnostic conclusions or etiological inferences. Reference ranges for quantitative measurements must be strictly validated against established medical knowledge databases. Clinical diagnoses should be synthesized through critical evaluation of the current medical history and the ophthalmologist's physical examination findings, with particular attention to pathognomonic signs and disease progression patterns. The diagnostic workflow should emphasize clinicopathological correlation and differential diagnosis exclusion criteria.

1. **Prompts of GPT-4o：**

This is a patient’s medical record. Using the history, ocular signs, and imaging, list all preliminary diagnoses (including but not limited to pseudophakia, refractive errors, and systemic diseases, with ICD coding), and provide a concise treatment plan in order of priority (specifying laterality for ocular interventions).

1. **Hyperparameter**

**GPT-4o**: API providers: OpenAI, version date: 2024-11-20, temperature=0.7, top_p=1.0, max_output_tokens=16,384 tokens.

**DeepSeek-R1 671B**: API providers: DeepSeek, version date: 2025-01-20, temperature=0.6, top_p=0.95, max_output_tokens=32,768 tokens.

**Appendix 2. Case Demonstration of ReasonAgent Application**

**eFigure 1**.The example input images for visual understanding module.

**
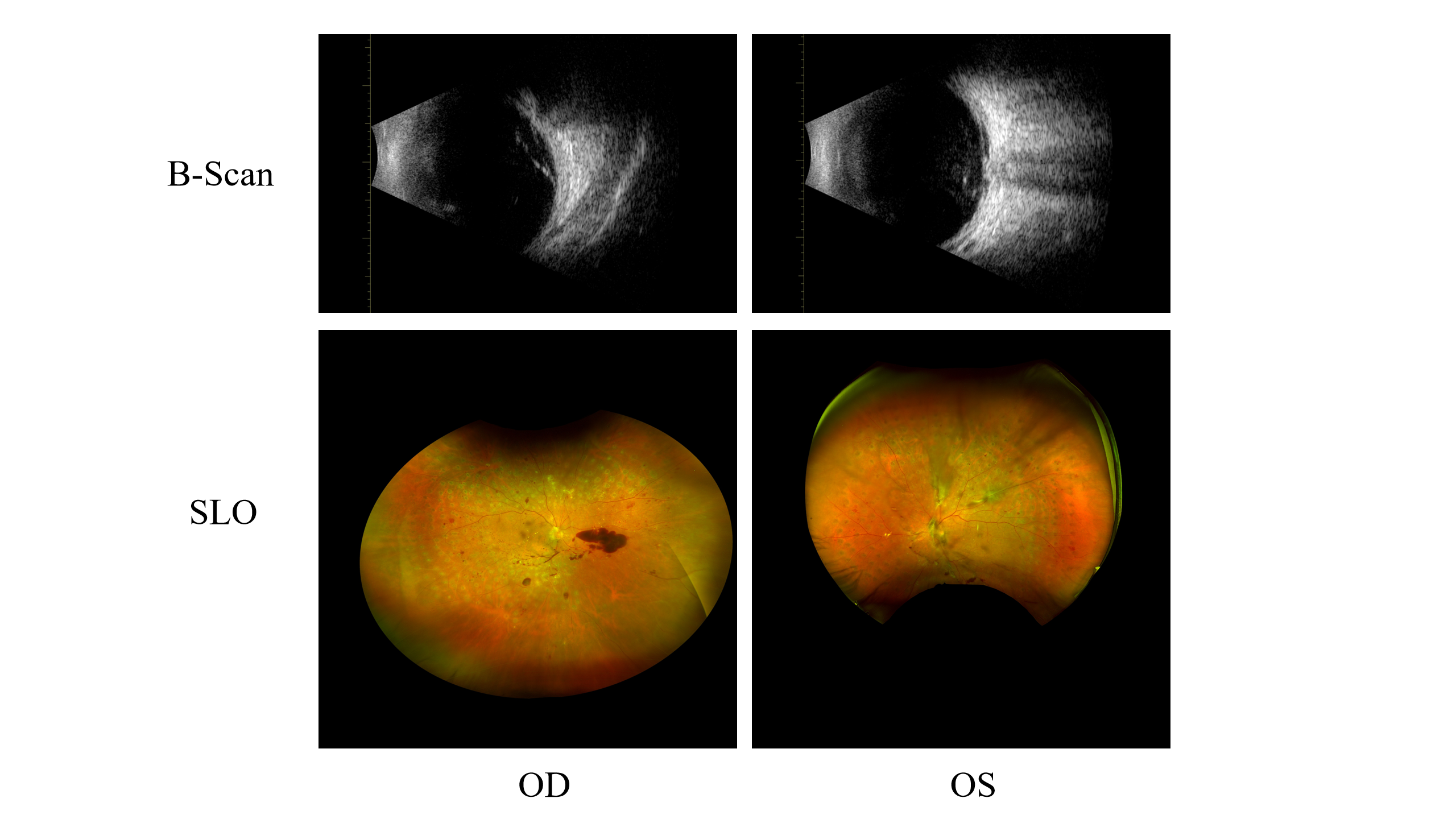
**

**Prompt:** The following is a patient's medical record. Based on the patient's medical history, ocular examination findings, and imaging modalities, please provide comprehensive preliminary diagnoses (including but not limited to pseudophakia, refractive errors, systemic diseases, etc., using ICD-11 international classification codes). Subsequently, formulate a prioritized management plan for the patient.

Special notes: The descriptive interpretations of imaging data are derived from GPT-4o's multimodal analytical capabilities for image recognition, which can be considered reliable for observational descriptions but should not be adopted for diagnostic conclusions or etiological inferences. Reference ranges for quantitative measurements must be strictly validated against established medical knowledge databases. Clinical diagnoses should be synthesized through critical evaluation of the current medical history and the ophthalmologist's physical examination findings, with particular attention to pathognomonic signs and disease progression patterns. The diagnostic workflow should emphasize clinicopathological correlation and differential diagnosis exclusion criteria.

A 54-year-old female with a history of diabetes (over 10 years, well-controlled with medication) and prior bilateral retinal laser photocoagulation (3 years ago) presents for "bilateral visual decline for over 6 months". The patient experienced gradual bilateral vision deterioration 6 months ago without identifiable triggers, with no ocular pain, redness, or other discomforts. She now seeks further evaluation. Exam: VA (uncorrected): OD 20/125, OS 20/100, BCVA (corrected): OD +1.75DS/-1.00DC ×73°→20/100, OS +1.25DS/-1.00DC ×80°→20/63. IOP (NCT): OD 12.0 mmHg, OS 11.0 mmHg. OD: Conjunctival non-injection, clear cornea, axial anterior chamber depth 4CT, clear aqueous, round pupil (3×3 mm) with positive light reflex, lens opacity (C2N2P1), mild vitreous opacity. Fundus exam: distinct optic disc margins with pale coloration, flat retina. The temporal posterior pole retina shows patchy hemorrhages and laser spots. OS: Conjunctival non-injection, clear cornea, axial anterior chamber depth 4CT, clear aqueous, round pupil (3×3 mm) with positive light reflex, lens opacity (C2N2P1), mild vitreous opacity. Fundus exam: distinct optic disc margins with pale coloration. A C-shaped proliferative membrane overlies the optic disc and superior vascular arcade, causing tractional grayish elevation of the superior retina. Extensive old laser spots were observed in the posterior pole.

The following are the imaging descriptive interpretations for the patient: Optical coherence tomography angiography (OCTA): In the right eye, interlayer punctate hyper-reflective signals can be observed. There is tractional elevation due to the proliferative membrane in the retina. Pre-retinal neovascularization is present, and areas of non-perfusion can be seen. Abnormal vascular anastomosis and capillary dilation are evident. In the left eye, cystoid macular edema is present. There is traction by a superficial proliferative membrane of the optic disc, accompanied by detachment of the neurosensory epithelial layer (tractional retinal detachment). Pre-retinal neovascularization is observable, and areas of non-perfusion are present. Abnormal vascular anastomosis is seen, capillary dilation is evident, and there are shielding signals (obscured by hemorrhage).**(The following descriptions were generated by the GPT-4o visual understanding module.)** Left eye B-scan imaging demonstrates high-echo reflective signals within the vitreous cavity, distributed in a patchy manner with high echo intensity, and partial echo signals exhibit a strong posterior shadowing effect, suggesting possible vitreous hemorrhage or inflammatory exudates. The globe structure is intact without obvious deformation or rupture. No significant signs of separation are observed in the retinal and choroidal regions, and typical features of retinal detachment are not indicated. The lens is in a normal position without obvious abnormal high-echo reflections. Additionally, no obvious intraocular mass or other abnormal structures are detected, with further diagnosis recommended by combining clinical history and other imaging findings. The right eye B-scan imaging also shows low-echo signals in the vitreous cavity presented as uneven fine punctate echoes, indicative of vitreous opacification or liquefaction. The posterior pole of the retina shows no obvious elevation or abnormal separation echoes, and characteristic high-echo signals of retinal detachment are absent, suggesting basic retinal attachment. The second image reveals mild echo enhancement of the posterior vitreous membrane, implying possible posterior vitreous detachment (PVD). No obvious intraocular mass or other abnormal high-echo signals are noted. For the OD eye, scanning laser ophthalmoscopy (SLO) shows a prominent dark red hemorrhage patch near the macular area with an irregular shape, likely resulting from subretinal or intraretinal hemorrhage. The optic disc is in a normal position, but its surrounding area appears slightly blurred, possibly affected by the lesion. Extensive laser photocoagulation scars are visible in the retinal mid-peripheral and peripheral regions, indicating a history of diabetic retinopathy or other retinal diseases requiring laser treatment. Overall, no obvious retinal detachment or break is observed in the images, though further clarification of the lesion nature and extent requires correlation with additional examinations such as optical coherence tomography (OCT) or fluorescein fundus angiography (FFA).

**DeepSeek-R1 reasoning process:** Comprehensive analysis of the 54-year-old female patient's medical history reveals a 10-year diabetes history with prior retinal laser photocoagulation. Ophthalmic examination demonstrates bilateral decreased corrected visual acuity (OD 0.2, OS 0.3) with normal intraocular pressures. Slit-lamp examination findings include lens opacity (C2N2P1 classification) and vitreous opacity in both eyes. Fundus examination reveals pale optic discs with temporal posterior pole hemorrhagic lesions and laser scars in the right eye, while the left eye exhibits C-shaped proliferative membrane traction, causing retinal detachment and extensive laser scars.

Ancillary examinations via Optical Coherence Tomography Angiography (OCTA) confirm bilateral neovascularization, non-perfusion areas, and capillary dilation, with additional macular cystic edema and tractional retinal detachment in the left eye. Ultrasonography identifies a suspected vitreous hemorrhage in the left eye and posterior vitreous detachment in the right eye. Scanning Laser Ophthalmoscopy (SLO) documents macular hemorrhage and pan-retinal laser photocoagulation scars in the right eye.

Diagnostic synthesis indicates proliferative diabetic retinopathy (PDR) with high-risk characteristics in the left eye, evidenced by tractional detachment and macular edema. The clinical presentation aligns with PDR diagnostic criteria, including neovascularization, fibrovascular proliferation, and vitreous hemorrhage. Management prioritizes urgent pars plana vitrectomy for left eye tractional detachment, followed by anti-VEGF therapy for bilateral macular edema and residual neovascularization. Elective cataract surgery should be deferred until retinal stabilization. Systemic optimization of glycemic control (target HbA1c <7%), blood pressure management (<130/80 mmHg), and lipid profile modulation complete the therapeutic protocol, consistent with current diabetic retinopathy management guidelines.

**DeepSeek-R1 output:**

Preliminary diagnosis：

1. Proliferative Diabetic Retinopathy (PDR), OU (ICD-10: E11.319)

OD: Retinal neovascularization, abnormal vascular anastomosis, non-perfusion areas, and vitreous hemorrhage (B-scan findings) ^1^.

OS: High-risk PDR (C-shaped fibrovascular proliferation with tractional retinal detachment, cystoid macular edema, and vitreous hemorrhage) ^2^.

1. Diabetic Macular Edema (DME), OS (ICD-10: E11.319) ^1, 3^.
2. Tractional Retinal Detachment, OS (ICD-10: H33.001) ^3^.
3. Age-related Cataract, OU (ICD-10: H25.9).
4. Refractive Error, OU (ICD-10: H52.9).
5. Type 2 Diabetes Mellitus with Ocular Complications (ICD-10: E11.39).

Treatment Plans:

1. Urgent surgical intervention for OS tractional retinal detachment: Pars Plana Vitrectomy (PPV) with membrane peeling, endolaser photocoagulation, and silicone oil/gas tamponade to relieve traction ^1, 2^.
2. Treatment for OS cystoid macular edema: Intravitreal anti-VEGF therapy (e.g., Ranibizumab) or corticosteroid implant (e.g., Dexamethasone) ^1, 3^.
3. OD PDR management: Panretinal Photocoagulation (PRP) or combined Anti-VEGF + PRP guided by FFA/ICGA to address neovascularization ^1, 2^.
4. Systemic control: multidisciplinary coordination with endocrinology for glycemic control (HbA1c <7%), blood pressure (<130/80 mmHg), and lipid profile (LDL-C <2.6 mmol/L) ^1, 4^.
5. Elective cataract surgery: Phacoemulsification with IOL Implantation once retinal stability is achieved.
6. Follow-up protocol: Postoperative monitoring: monthly OCT, intraocular pressure (IOP), and fundus examination. Non-surgical eye: quarterly OCT and DR progression assessment (adjust frequency based on DME activity) ^1, 4^.

Reference：

Superscripts 1-4 are all cited from the *Chinese Clinical Diagnosis and Treatment Guidelines for Diabetic Retinopathy (2022 Version)*, covering diagnostic criteria, staging, therapeutic strategies, and systemic management recommendations for PDR/DME.

**Appendix 3. Ablation Study**

**The effectiveness analysis of GPT-4o**: As shown in eTable 1, exclusion of GPT-4o (w/o GPT-4o) led to diagnostic errors due to the unavailability of imaging data, manifesting as misdiagnosis of proliferative retinopathy OD with ambiguous diagnostic descriptions (e.g., likely diabetic retinopathy, advanced stage). This highlights the critical role of visual data understanding in ophthalmic diagnostics.

**The effectiveness analysis of DeepSeek-R1**: As shown in eTable 1, replacement of DeepSeek-R1 with GPT-4o (w/o Deepseek-R1) caused a significant reduction in diagnostic specificity, exemplified by confusion between Coats disease and diabetic retinopathy. This discrepancy likely stems from DeepSeek-R1's specialized optimization in clinical causal reasoning (including disease staging and pathological logic derivation), whereas general-purpose LLMs like GPT-4o tend to make probabilistic associations (e.g., erroneously attributing retinal exudates to metabolic disorders).

**The effectiveness analysis of RAG**: As shown in eTable 1, the experiment confirms RAG's clinical enhancement effect on treatment planning. Absence of RAG (w/o RAG) resulted in standardized therapeutic deviations, particularly through overly generic recommendations (e.g., proposing OCT/MRI and pars plana vitrectomy without prerequisite evaluations like laser therapy failure assessment or retinal detachment analysis), indicating compromised alignment with evidence-based clinical guidelines.

These findings collectively validate the synergistic value of the vision-knowledge-reasoning integration mechanism. The ReasonAgent framework achieves refined hierarchical diagnosis, while the absence of any component may lead to diagnostic inaccuracies or inadequately contextualized treatment plans.

**eTable 1**. **Comparative Diagnostic and Treatment planning Outputs under Component Exclusion Scenarios**

|  | ReasonAgent | w/o RAG | **^*^**w/o Deepseek-R1 | w/o GPT-4o |
| --- | --- | --- | --- | --- |
| Diagnosis | 1. **Coats disease OD** 2. Exudative retinal detachment OD 3. Retinal neovascularization OD | 1. **Coats disease OD** 2. Exudative retinal detachment OD 3. Proliferative retinopathy OD 4. Vitreous opacity OD | 1. **Proliferative Retinopathy OD (likely diabetic retinopathy, advanced stage)** 2. Vitreous Opacity OD 3. Normal OS 4. Possible systemic metabolic disorder (diabetes mellitus) | 1. **Familial Exudative Vitreoretinopathy (FEVR) OD** 2. Secondary Retinal Detachment OD 3. Retinal Neovascularization OD |
| Treatment  Plans | 1. Right eye anti-VEGF therapy combined with retinal laser photocoagulation. 2. Systemic metabolic and genetic disorder screening. 3. Regular fundus follow-up of the right eye (every 1-2 months). | 1. Complete right eye OCT and ocular MRI examination. 2. Right eye anti-VEGF therapy combined with retinal laser photocoagulation. 3. Comprehensive pediatric evaluation and genetic metabolic screening. 4. Left eye follow-up examination every 3 months. 5. Right eye retinal reattachment surgery if necessary. | 1. Right eye anti-VEGF therapy combined with retinal laser photocoagulation. 2. Close follow-up of right eye fundus status with monthly ophthalmic examinations (including FFA and OCT) 3. Comprehensive systemic evaluation including fasting blood glucose, HbA1c, lipid profile, and renal function tests 4. Pars plana vitrectomy (PPV) if disease progression occurs | 1. Fundus fluorescein angiography (FFA) and optical coherence tomography (OCT) of the right eye 2. Intravitreal injection of anti-vascular endothelial growth factor (VEGF) agents in the right eye 3. Focal retinal laser photocoagulation targeting non-perfusion areas and neovascularization in the right eye 4. Genetic counseling and comprehensive systemic evaluation |

**w/o DeepSeek-R1 replaces the reasoning module with GPT-4o while maintaining RAG integration.*

*RAG: Retrieval-Augmented Generation; OD: Right Eye; OS: Left Eye*
